# Frontiers in Systemic Therapy for Unresectable or Metastatic Adrenocortical Carcinoma: Harnessing Novel Therapeutic Approaches

**DOI:** 10.1177/11795549251364042

**Published:** 2025-08-29

**Authors:** Adam E Singer, Nikhita Kathuria-Prakash, David Shabsovich, Lizette Garcia, Leland Damron, Allan J Pantuck, Alexandra Drakaki

**Affiliations:** 1 Division of Hematology/Oncology, Department of Internal Medicine, University of California, Los Angeles, Los Angeles, CA, USA; 2 Department of Internal Medicine, University of California, Los Angeles, Los Angeles, CA, USA; 3 Department of Urology, University of California, Los Angeles, Los Angeles, CA, USA

**Keywords:** Adrenocortical carcinoma, systemic therapy, tyrosine kinase inhibitors, immune checkpoint inhibitors, radiopharmaceuticals

## Abstract

Adrenocortical carcinoma is a rare, aggressive endocrine malignancy with limited effective systemic therapy options and an overall poor prognosis for unresectable or metastatic disease. Chemotherapy and mitotane are the traditional systemic therapies of choice. More recently, as drug development in oncology has shifted away from chemotherapy, a host of therapeutic approaches targeting novel mechanisms of action has been studied in adrenocortical carcinoma. This review summarizes all nonchemotherapeutic approaches that have been and/or are currently being tested in clinical trials for the treatment of unresectable or metastatic adrenocortical carcinoma.

## Introduction

Adrenocortical carcinoma (ACC) is a rare, aggressive endocrine malignancy with an annual incidence of approximately one per million.^1^ Nearly half of patients have nodal or distant metastases at the time of presentation.^2^ Early-stage disease can be treated with surgical resection, which is potentially curative, but the disease frequently relapses after initial resection. Prognosis for unresectable or metastatic disease is very poor, with a median overall survival (OS) of approximately 14 months.^1,3^

The mainstay of systemic therapy for unresectable or metastatic disease has traditionally been chemotherapy with or without the adrenolytic agent mitotane. The 2012 phase III FIRM-ACT trial definitively established the combination of etoposide, doxorubicin, and cisplatin (EDP) plus mitotane as the preferred first-line option for fit patients.^3^ Even with this intensive combination regimen, however, objective response rate (ORR) was only 23%, median progression-free survival (PFS) 5 months, and median OS 14.8 months. A host of less intensive chemotherapy regimens has been evaluated in smaller studies over the last several decades (Table 1).^4-15^ These demonstrate modest efficacy as a whole and are typically reserved for later lines of therapy or for patients who cannot tolerate EDP-mitotane.

**Table 1.** Chemotherapeutic strategies for advanced adrenocortical carcinoma.

Study (year)	Study design	Trial phase	Treatment regimen	N	Line	ORR (%)	DCR (%)	mPFS (months)	mDOR (months)	mOS (months)	Notes	Reference
Decker et al	Prospective, 2-arm, nonrandomized	II	Mitotane	36	1L or 2L	22.2	NR	NR	NR	14.0	Patients in the doxorubicin arm had progressed on mitotane or had poor tumor prognostic markers	4
			Doxorubicin	16		18.8	NR	NR				
Bonacci et al	Prospective, single-arm	II	Cisplatin, Etoposide ± Mitotane	18	2L	33.3	44.4	NR	NR	NR	1L was mitotane, and 14 patients continued mitotane during study	5
Williamson et al	Prospective, single-arm	II	Cisplatin, Etoposide followed by Mitotane	45	1L or 2L	11.1	NR	NR	NR	10.0	If 2L, 1L was single-agent mitotane	6
Baudin et al	Prospective, single-arm	II	Irinotecan	12	2L or later	0	25.0	NR	1.5	NR	In 3 patients, 1L was single-agent mitotane	7
Berruti et al	Prospective, single-arm	II	Etoposide, Doxorubicin, Cisplatin, Mitotane	72	1L	48.6	76.4	9.1	NR	28.5		8
Sperone et al	Prospective, single-arm	II	Gemcitabine, Mitotane, 5-fluorouracil or Capecitabine	28	2L or 3L	7.1	46.4	5.3	6.0	9.8		9
Berruti et al	Prospective, single-arm	II	Paclitaxel, Sorafenib	10	2L or 3L	0	0	*Trial terminated early*				10
Fassnacht et al	Prospective, 2-arm, randomized	III	Etoposide, Doxorubicin, Cisplatin, Mitotane	151	1L	23.2	58.3	5.0	NR	14.8	Prior mitotane was allowed. Upon disease progression or unacceptable toxicity, patients crossed over to the other study arm.	3
			Streptozosin, Mitotane	153		9.2	31.4	2.1	NR	12.0		
Urup et al	Prospective, single-arm	II	Docetaxel, Cisplatin	19	1L	21.1	52.6	3.0	NR	12.5		11
Kroiss et al	Retrospective case series	N/A	Trofosfamide ± Mitotane	21	1L or later	0	14.3	2.8	NR	6.5	14 patients received mitotane	12
Kroiss et al	Retrospective case series	N/A	Thalidomide, Mitotane	27	2L or later	0	7.4	2.6	NR	8.4		13
Cosentini et al	Retrospective case series	N/A	Temozolomide ± Mitotane	28	2L or later	21.4	35.7	3.5	NR	7.2	12 patients received mitotane	14
Laganà et al	Prospective, single-arm	II	Cabazitaxel	25	2L or later	0	24.0	1.5	NR	6.0		15

RR = response rate; DCR = disease control rate; PFS = progression-free survival (reported as median months); OS = overall survival (reported as median months); CT = chemotherapy; NR = not reported; 1 L = first-line; 2 L = second-line; 3 L = third-line.

The only US Food and Drug Administration (FDA)-approved systemic therapy for ACC is mitotane, and there is a paucity of compendia-listed treatment options.^16,17^ Additional effective systemic therapies are needed to combat this lethal disease. Accordingly, there has been immense interest in harnessing novel mechanisms of action informed by preclinical work to expand the menu of treatment options, with many studies completed and many more ongoing.

In this narrative review, we summarize the evidence for nonchemotherapeutic treatment approaches to unresectable or metastatic ACC that have already been tested in clinical studies. Two key areas are not included in this review: (1) biochemical pathways of potential therapeutic interest that have been evaluated only in preclinical studies and (2) approaches expected to be active in ACC tumors harboring specific sensitizing genomic alterations found only in subsets of ACC (e.g., antihuman epidermal growth factor receptor 2 [HER2] agents for ACC tumors that overexpress HER2).

## VEGFR TKIs

Tyrosine kinase inhibitors (TKIs) targeting vascular endothelial growth factor (VEGF) receptor (VEGFR), such as sunitinib, sorafenib, pazopanib, regorafenib, axitinib, cabozantinib, lenvatinib, and tivozanib, have transformed the treatment of renal cell carcinoma (RCC), hepatocellular carcinoma (HCC), and thyroid cancer, among others. In ACC, angiogenesis is thought to play a key role in tumor growth and survival, as evidenced by the influence of vasculogenic mimicry in tumor growth, overexpression of VEGFR on the surface of tumor cells, and elevated serum levels of VEGF in patients with ACC.^18,19^ Several completed and 2 ongoing trials have sought to assess the efficacy of VEGFR TKIs in treating unresectable or metastatic ACC.

Sorafenib, sunitinib, and axitinib were the first 3 VEGFR TKIs studied in ACC, all in prospective, phase II trials. Berruti et al^10^ studied sorafenib in combination with weekly paclitaxel in 25 patients with metastatic ACC who progressed after mitotane and 1 or 2 chemotherapies, with a primary endpoint of PFS. The trial was stopped prematurely at the time of first radiologic evaluation due to disease progression in all evaluable patients. Kroiss et al^20^ studied sunitinib in 38 patients with metastatic ACC who progressed after mitotane and 1 to 3 cytotoxic chemotherapies, including a platinum-based chemotherapy regimen, with a primary endpoint of PFS. Five of 35 evaluable patients (14%) had stable disease (SD), while the remainder had progressive disease (PD) or died before the first evaluation of disease status. The median PFS was 2.8 months, but in responders the PFS ranged between 5.6 and 11.2 months and OS ranged between 14.0 and 35.0 months. O’Sullivan et al^21^ studied axitinib in 13 patients with metastatic ACC as a second-line therapy after chemotherapy, with a primary endpoint of PFS. At first interim analysis, all patients had PD, although the rate of growth was slowed in 4 patients.

Cabozantinib is currently under investigation as a treatment option for refractory ACC. In a retrospective cohort study by Kroiss et al, 16 patients received off-label cabozantinib after mitotane and an average of 3 additional lines of therapy. Three patients achieved a partial response (PR), and 5 patients had SD, with a median PFS of 16 weeks.^22^ Given this promising data, 2 prospective, phase II trials of cabozantinib are underway (Table 2).^23,24^

**Table 2.** Ongoing prospective therapeutic trials in unresectable or metastatic adrenocortical carcinoma.

Compound	Mechanism of Action	Disease	Trial Acronym	Trial Phase	Trial Design	Randomized	Comparator	Line of Therapy	Patient Enrollment Target	Country	Expected Completion	Reference
Cabozantinib	VEGFR TKI	ACC	-	II	Single-arm	-	-	2L and later	18	USA	Completed 2024, awaiting results	^23^
Cabozantinib	VEGFR TKI	ACC	CaboACC	II	Single-arm	-	-	2L and later	37	Germany	2025	^24^
Dostarlimab	ICI (single-agent)	MSI-H solid tumors including ACC	-	II	Two-arm	Yes	Chemotherapy	1L	120	France	2030	^39^
Pembrolizumab	ICI (single-agent)	ACC	PEMBR-01	II	Single-arm	-	-	2L	24	Poland	2027	^40^
Ipilimumab and nivolumab	ICI (dual)	Rare genitourinary malignancies including ACC	-	II	Single-arm	-	-	2L and later	100	USA	2025	^41^
Ipilimumab and nivolumab	ICI (dual)	Rare malignancies including ACC	DART	II	Two-arm	No	Nivolumab	2L and later	818	USA	2026	^42^
Cabozantinib and atezolizumab	VEGFR TKI and ICI	ACC	-	II	Single-arm	-	-	1L and later	21	USA	2025	^48^
Lenvatinib and pembrolizumab	VEGFR TKI and ICI	ACC	ACCOMPLISH	II	Single-arm	-	-	2L and later	30	Korea	2026	^49^
ADCT-701	ADC	Neuroendocrine malignancies including ACC	-	I	Single-arm	-	-	2L and later	70	USA	2029	^55^
Megestrol acetate and EDP-mitotane	Progesterone analogue and chemotherapy	ACC	PESETA	II	Two-arm	Yes	EDP-mitotane	1L	80	Italy	2027	^87^
Relacorilant and pembrolizumab	Glucorticoid receptor modulator and ICI	ACC	-	Ib	Single-arm	-	-	1L and later	15	USA	Completed 2024, awaiting results	^94^
mRNA-0523-L001	mRNA neoantigen vaccine	Endocrine malignancies including ACC	-	N/A	Single-arm	-	-	1L and later	21	China	2025	^102^
B7 H3 CAR-T cells	Autologous CAR-T cells	Solid tumors including ACC	3CAR	I	Single-arm	-	-	2L and later	32	USA	2027	^106^

Abbreviations: ADC, antibody-drug conjugate; CAR-T, chimeric antigen receptor T-cell therapy; EDP, etoposide, doxorubicin, and cisplatin; ICI, immune checkpoint inhibitor. MSI-H, microsatellite instability high; TKI, tyrosine kinase inhibitor; VEGFR, vascular endothelial growth factor receptor; 1L, first-line; 2L second-line.

A key consideration in using VEGFR TKIs in ACC is that all VEGFR TKIs are metabolized by CYP3A4 and mitotane is a strong CYP3A4 inducer, leading to significant drug interactions with all VEGRF TKIs except lenvatinib. Mitotane’s half-life can be up to 5 months long, and it is often continued during all lines of therapy for ACC, introducing a practical problem of how a VEGFR TKI might be studied (and utilized) effectively. In Kroiss et al,^20^ mitotane was stopped just prior to enrollment, and several patients still had elevated plasma mitotane levels while on study. Blood levels of sunitinib and its active metabolite were measured and the median concentration was 29.3 ng/mL, far below the known threshold of 50 ng/mL required for sunitinib therapeutic activity. The 2 ongoing cabozantinib trials have incorporated this lesson and require a plasma mitotane concentration of less than 2 mg/L prior to study enrollment.^23,24^

Notably, however, Berruti et al^10^ required a 1-month washout period between mitotane discontinuation and sorafenib initiation, and mitotane levels were low in most patients. This study still failed to demonstrate activity of sorafenib in ACC, suggesting that subtherapeutic exposure to VEGFR TKIs is not the only factor driving tumor resistance to this mechanism of action. Additional research is needed to understand the underlying reasons for this resistance and why a small subset of patients has tumors that appear to be sensitive to VEGFR TKIs.

## Immune Checkpoint Inhibitors

Immune checkpoint inhibitors (ICIs) targeting programmed cell death 1 (PD-1), programmed cell death ligand 1 (PD-L1), and cytotoxic T-lymphocyte antigen 4 (CTLA-4) have revolutionized the treatment of many malignancies, and have been of great interest in ACC. Two retrospective observational studies suggested improved PFS when ICI was administered in combination with chemotherapy and mitotane,^25,26^ leading to a host of prospective immunotherapy trials in ACC.

Le Tourneau et al^27^ studied avelumab with or without mitotane in a prospective, phase Ib trial in 50 patients who had received at least 2 prior lines of therapy. Three patients (6%) had PR and an additional 21 patients (42%) had SD. The median PFS was 2.6 months. Carneiro et al^28^ studied nivolumab as a second-line therapy in a prospective, phase II trial of 10 patients. Two patients had SD (20.0%) and the remainder had PD after a median of 2 doses, with a median PFS of 1.8 months.

Pembrolizumab has been evaluated in 2 independent prospective trials. Habra et al^29^ studied pembrolizumab in a prospective, phase II trial in patients with rare malignancies, including 16 patients with ACC, of whom 14 were evaluable. Two patients had PR (14%) and 7 had SD (50%), for a disease control rate (DCR) of 64%. Raj et al^30^ studied pembrolizumab in a prospective, phase II trial in 39 patients with ACC. Nine patients (23%) had PR and 7 had SD (18%), for a DCR of 41%. Notably, pembrolizumab with or without mitotane is NCCN-listed for unresectable or metastatic ACC.^17^

Klein et al^31^ studied combination ipilimumab and nivolumab in patients with rare malignancies, including 6 patients with ACC. Two patients had PR (33%) and 2 patients had SD (33%), for a DCR of 66%.

Immune checkpoint inhibitor has demonstrated enhanced efficacy in tumors with high microsatellite instability (MSI-H) or high tumor mutational burden (TMB high), resulting in tumor-agnostic FDA approvals for pembrolizumab in patients with MSI-H or TMB high tumors who have progressed on prior treatment and have no satisfactory alternative options.^32,33^ ACC is a Lynch syndrome-associated cancer, with 3.2% of ACCs demonstrating mutations in mismatch repair.^34^ Two of the above trials of ICI in ACC stratified patients by MSI-H status in subgroup analyses. In the Klein et al^31^ study of combination ipilimumab and nivolumab, both patients who had PR had MSI-H tumors, and notably these responses appeared durable, lasting for over 10 and 25 months, respectively. In the Raj et al^30^ study of pembrolizumab, 6 patients had MSI-H tumors, of whom 2 (33%) had PR and 2 (33%) had SD at 24 months. These results suggest that ICI may be more effective in MSI-H than microsatellite stable (MSS) ACC tumors and may produce more durable responses.

Approximately half of ACC tumors secrete glucocorticoids, and excess cortisol can lead to immune suppression and theoretically counteract the efficacy of ICI. Three of the above trials stratified patients by cortisol-producing status in subgroup analyses. In the Carneiro et al^28^ study of nivolumab, 2 of 10 patients had cortisol-producing tumors and had PD. In the Klein et al^31^ study of combination ipilimumab and nivolumab, 2 of 6 patients had cortisol-producing tumors. One patient (50%) had an MSS tumor and had SD; the other (50%) had an MSI-H tumor and had PR. In the Habra et al^29^ study of pembrolizumab, 7 of 16 patients had cortisol-producing tumors. One (14%) had PR and 3 (43%) had SD, similar to the efficacy observed in the overall study population. Overall, these results suggest that ICI may retain efficacy in cortisol-producing ACC tumors.

Taken as a whole, these prospective ICI studies clearly demonstrate activity of ICI in some cases of unresectable or metastatic ACC. Although response rates are low overall, SD rates tend to be higher, leading to reasonably high DCRs. Nevertheless, ACC appears less responsive to immunotherapy than some other tumor types such as melanoma, nonsmall cell lung cancer (NSCLC), and RCC. The mechanisms underlying this are likely multifactorial: studies have suggested low TMB, low cancer stemness indices, molecular alterations that result in T-cell exclusion, and a tumor microenvironment that supports immune evasion in the setting of hypercortisolism as possible reasons for decreased sensitivity to ICI in ACC.^35-38^

Additional prospective trials of dostarlimab in MSI-H ACC and pembrolizumab and combination ipilimumab and nivolumab in MSI-H or MSS ACC are underway (Table 2).^39-42^ In addition, with inhibition of novel checkpoints such as lymphocyte-activation gene 3 (LAG3) and T-cell immunoreceptor with immunoglobulin and immunoreceptor tyrosine-based inhibition motif domain (TIGIT) under investigation across tumor types, and the FDA approval of combination nivolumab and the anti-LAG3 checkpoint inhibitor relatlimab for unresectable or metastatic melanoma,^43^ novel checkpoint inhibitors may be investigated in ACC in the future.

## Combination VEGFR TKIs and ICI

More recently, attention has shifted to the use of combination VEGFR TKIs and ICI in unresectable or metastatic ACC. Such combinations are the mainstay of treatment in RCC and HCC, and are used in the certain treatment scenarios in some other tumor types as well. There is thought to be mechanistic synergy between the 2 drug classes: VEGF promotes cancer immune evasion through a variety of mechanisms, and inhibition of VEGFR can normalize abnormal tumor vasculature, allowing for increased infiltration of immune effector cells into tumors and the resultant conversion of the tumor microenvironment from an immunosuppressive to an immunosupportive one.^44^

Bedrose et al^45^ reported a retrospective case series of combination lenvatinib and pembrolizumab as salvage therapy for heavily pretreated metastatic ACC. Eight patients were treated with this combination after a median of 4 prior lines of therapy, including prior VEGFR TKI and ICI monotherapies. Two patients (25%) had PR and 1 patient (12.5%) had SD, with a median PFS of 5.5 months.

Two prospective trials of combination VEGFR TKI and ICI have been completed to date. Grande et al^46^ studied combination cabozantinib and atezolizumab in a prospective, phase II trial of advanced and refractory endocrine and neuroendocrine tumors in 6 cohorts, one of which was ACC, which enrolled 24 patients. Patients in the ACC cohort had disease progression on at least one prior line of therapy, and 13 of 24 (54%) had received at least 2 prior lines of therapy. Two patients (8.3%) had PR that lasted for 5.4 and 17.4 months, while the remainder of patients had PD. Median PFS was 2.9 months. Mitotane discontinuation status and MSI-H status were not reported, and results were not stratified by tumor cortisol-producing status.

Wei et al^47^ studied combination apatinib and camrelizumab, a VEGFR TKI and PD-1 inhibitor, respectively, under investigation in China for a variety of malignancies, as second-line therapy in 21 patients with unresectable or metastatic ACC. Eleven patients (52%) had PR and 9 patients (43%) had SD, for a DCR or 95%. Median PFS was 12.6 months and median OS was 20.9 months. Eight of 21 patients had cortisol-producing tumors; of these, 2 (25%) had PR, 5 (63%) had SD, and 1 (13%) had PD. Two of 21 patients had MSI-H tumors, neither of which were cortisol-producing; of these, 1 (50%) had PR and 1 (50%) had SD. Mitotane discontinuation status was not reported. In exploratory analyses, a higher baseline CD8-positive T-cell percentage and peripheral blood CD4- and CD8-positive T cell abundance were both associated with treatment response.

The results of Wei et al stand in contrast to those of Grande et al. This may have been partially influenced by study design: all patients in Wei et al^47^ had received only one prior line of therapy, while just over half of patients in Grande et al^46^ had received at least 2 prior lines of therapy. Ultimately, though, the underlying reasons for the disparate outcomes in these 2 studies are currently unclear. At the time of writing of this review, these 2 studies have been published in abstract form only; full publications may shed more light on their results.

Two additional prospective trials of combination VEGFR TKI and ICI in unresectable or metastatic ACC are underway: a second trial of cabozantinib and atezolizumab and a trial of lenvatinib and pembrolizumab (Table 2).^48,49^

## Antibody-Drug Conjugates

Antibody-drug conjugates (ADCs) consist of a monoclonal antibody linked to a cytotoxic payload, usually a chemotherapeutic agent. The monoclonal antibody is designed to recognize an antigen present on tumor cells. When the ADC binds to its target antigen, it releases its payload directly to the tumor cell. Successful ADCs target antigens present in high concentrations on tumor cells and low concentrations on normal tissues, which maximizes therapeutic drug delivery and minimizes adverse effects. Many ADCs are FDA-approved for the treatment of a variety of malignancies.^50^

There is emerging interest in using ADCs to treat unresectable or metastatic ACC. Identifying antigen candidates has been a challenge, as ACC has among the least potential overexpressed targets of any cancer.^51,52^ Nevertheless, 3 ADCs with unique targets have been developed to date.

Lemech et al^53^ studied ABBV-176, an ADC targeting the prolactin receptor linked to a cytotoxic pyrrolobenzodiazepine (PBD) dimer, in a phase I trial that enrolled 19 patients with various tumor types, including 2 with ACC. There were no objective responses and patients experienced significant toxicities with ABBV-176, leading to study termination. Another ADC, ADCT-701, which targets DLK1, a Notch ligand, linked to a PBD dimer, demonstrated efficacy in preclinical ACC xenografts, and a phase I trial is underway (Table 2).^54,55^ A third ADC, ADCT-211, which targets interleukin 13 receptor subunit alpha 2 (IL13RA2) linked to a PBD dimer, has shown antitumor activity in *in vitro* and *in vivo* studies, and a phase I trial is expected.^56^

Clinical data from studies of ADCs in ACC remain largely immature, and it remains to be seen whether this class of drugs will have efficacy in treating ACC.

## IGF Pathway Inhibition

The insulin-like growth factor (IGF) pathway consists of 3 receptors with corresponding ligands which are crucial for cellular signaling and proliferation. This pathway has been shown to be upregulated in 90% of ACC tumors, which has led to investigation into its therapeutic targeting.^57^

Three single-arm trials of IGF 1 receptor (IGF-1R) inhibitors in ACC have been completed. Haluska et al^58^ studied figitumumab, a monoclonal antibody targeting IGF-1R, in a phase I trial of 14 patients with progressive metastatic ACC. There were no objective responses. Six patients (43%) had SD at 3 months, and all patients discontinued figitumumab by 5 months due to PD or adverse events. Naing et al^59^ studied cixutumumab, a monoclonal antibody targeting IGF-1R, in combination with the mammalian target of rapamycin (mTOR) inhibitor temsirolimus in a phase I trial of 26 patients with metastatic ACC. Patients were heavily pretreated, with a median of 4 prior therapies. There were no objective responses, but 11 patients (42%) had SD for at least 6 months. Lerario et al^60^ studied cixutumumab in combination with mitotane in a prospective, single-arm trial of 20 patients with metastatic ACC in the first-line setting. One patient (5%) had PR and 7 patients (35%) had SD, while the remainder progressed. The median PFS was only 6 weeks. This study was ultimately terminated due to poor accrual and limited efficacy.

Fassnacht et al^61^ studied linsitinib, a small molecule inhibitor of IGF-1R, in a phase III trial of 139 patients with progressive metastatic ACC. Patients were randomized to linsitinib or placebo. There was no difference in OS (the primary endpoint) or PFS between the arms. Interestingly, 3 patients in the linsitinib arm (3%) had PR lasting for several years each. All 3 patients had low-grade ACC with Ki-67 ⩽ 20%. There was also a trend toward higher DCR in the linsitinib arm. Nevertheless, this study did not meet its primary endpoint and was a negative trial.

Despite preclinical work showing the importance of the IGF pathway in ACC, clinical studies did not show consistent benefit, and no further efforts are underway to target the IGF pathway in ACC.

## FGFR Pathway Inhibition

The fibroblast growth factor receptor (FGFR) pathway is involved in several key cellular functions. ACC tumors have been shown to harbor a number of alterations in FGFR signaling, prompting investigation into therapeutic targeting of this pathway.^62^

García-Donas et al^63^ studied dovitinib, a TKI that inhibits multiple targets including FGFR1/3, in a prospective, phase II trial of 17 patients with unresectable or metastatic ACC. Prior therapy only with mitotane was allowed. One patient (6%) had PR and 4 patients (23%) had SD lasting at least 6 months, with a median PFS of 1.8 months. This study did not meet its primary endpoint of ORR and was a negative trial. Papadopoulos et al^64^ studied derazantinib, a TKI that inhibits multiple targets including pan-FGFR, in a phase I trial of 80 patients, including 4 patients with ACC. One patient with an ACC tumor harboring an FGFR1 amplification had SD and was on study for 3.5 years. A second patient with an ACC tumor without FGFR amplifications had SD and was on study for at least 12 months.

Neither dovitinib nor derazantinib is FDA approved, and to our knowledge further testing of these drugs in ACC has halted. Since the above trials were conducted, however, the FDA has approved 4 other FGFR inhibitors for other disease indications: the pan-FGFR inhibitors erdafitinib and futibatinib, and the FGFR1/2/3 inhibitors pemigatinib and infigratinib (accelerated approval for infigratinib was later voluntarily withdrawn by the manufacturer due to poor enrollment on the confirmatory trial).^65,66^ None of these agents has been tested in ACC. Given the potentially encouraging signals in García-Donas et al and Papadopoulos et al, notably the long duration of SD observed in some patients, these newer generation FGFR inhibitors may hold promise if formally trialed in ACC.

## EGFR Pathway Inhibition

Epidermal growth factor receptor (EGFR) plays an important role in normal epithelial cell physiology and is overexpressed or mutated in many cancers, promoting tumorigenesis. Epidermal growth factor receptor driver mutations, most notably in NSCLC, confer sensitivity to EGFR inhibitors, while EGFR overexpression typically does not.^67,68^ In ACC, over 75% of tumors express EGFR, but mutations in EGFR are rare.^69^

Two trials of EGFR pathway inhibition in ACC have been completed. Samnotra et al^70^ studied the first-generation EGFR inhibitor gefitinib as monotherapy in a prospective, phase II trial of 19 patients with progressive ACC. All patients had PD. Quinkler et al^71^ studied the first-generation EGFR inhibitor erlotinib in combination with gemcitabine in a prospective trial of 10 patients with progressive ACC after 2 to 4 prior lines of therapy. One patient (10%) had SD that lasted for 8 months, while the remainder had PD. Of note, the patient with SD had an ACC tumor with the lowest EGFR expression of any of the 10 patients.

Epidermal growth factor receptor pathway inhibition in ACC has yielded disappointing results, likely owing to the low rate of EGFR mutations in ACC, and EGFR inhibitors are no longer being investigated in ACC.

## PI3K/AKT/mTOR Pathway Inhibition

The phosphoinositide 3-kinase (PI3K)/AKT/mTOR signaling pathway is involved in cellular metabolism and growth and has been implicated in ACC tumorigenesis. Preclinical studies have demonstrated potential efficacy of mTOR inhibitors in human ACC cell lines.^72^

Fraenkel et al^73^ studied the mTOR inhibitor everolimus as monotherapy in 4 patients with progressive ACC. All 4 patients had PD 1 to 4 months after starting therapy. Ganesan et al^74^ studied the mTOR inhibitor temsirolimus in combination with lenalidomide in a phase I trial of 43 patients with advanced cancer, including 3 patients with ACC. One patient (33%) had SD for at least 6 months. As noted above, Naing et al^59^ studied the mTOR inhibitor temsirolimus in combination with the IGF-1R inhibitor cixutumumab in a phase I trial of 26 patients with metastatic ACC. Patients were heavily pretreated, with a median of 4 prior therapies. There were no objective responses, but 11 patients (42%) had SD for at least 6 months.

More recently, Hong et al^75^ studied eganelisib, a first-in-class PI3K-γ inhibitor, as monotherapy or with nivolumab, in a phase I trial of 219 patients with progressive advanced cancer, including 6 patients with ACC. All ACC patients received combination eganelisib and nivolumab. One patient (17%) had PR that lasted 11.4 months. The remaining 5 patients did not have an objective response, but their best response (SD versus PD) was not reported.

While most of the patients treated with PI3K or mTOR inhibitors in these trials had PD, some patients experienced disease control, highlighting the need for further investigation into the PI3K/AKT/mTOR pathway in treating ACC.

## SOAT1 Inhibition

Sterol-O-acyl transferase 1 (SOAT1), also known as acyl-coenzyme A: cholesterol acyltransferase 1 (ACAT1), is involved in cholesterol processing in the adrenal cortex.^76^ In preclinical studies, mitotane has been shown to inhibit SOAT1, leading to accumulation of toxic lipids and subsequent apoptosis of ACC cells.^77^ As a result, SOAT1 was initially investigated as a prognostic marker in ACC but showed mixed results.^77-79^ More recently, Smith et al^80^ studied the adrenal-specific SOAT1 inhibitor nevanimibe in a phase I trial of 63 patients with progressive metastatic ACC. There were no objective responses, but 13 of 48 evaluable patients (27%) had SD at 2 months and 4 patients (8%) had SD at 4 months. Of note, very few dose-limiting toxicities occurred, a maximum tolerated dose could not be defined, and expected exposure levels necessary to achieve apoptosis were not reached, which may have contributed to the limited efficacy of nevanimibe in this study.

There are no other clinical studies of SOAT1 inhibition in ACC ongoing at this time, but several other compounds have been shown to target SOAT1 in preclinical studies, including the SOAT1 inhibitor avasimibe and the BCR-ABL TKI nilotinib.^81^ These may be of interest in future clinical studies in ACC.

## Progesterone

Abiraterone acetate (AA) is a potent inhibitor of 17α-hydroxylase/17,20-lyase, a key enzyme in adrenal steroidogenesis, which may help suppress excess cortisol from steroid-secreting ACC tumors. Preclinical studies investigating the effect of AA on ACC cell lines also demonstrated a cytotoxic effect that is thought to be mediated by a drug-induced increase in progesterone levels.^82^ Increased progesterone levels in turn lead to activation of progesterone receptors, which alter cellular programs via their classical action as nuclear transcription factors as well as so-called extranuclear effects. Several subsequent preclinical studies demonstrated a cytotoxic effect of progesterone itself, alone or in combination with ribociclib or tamoxifen, on a variety of ACC cell lines.^83-86^

Based on this preclinical work, a prospective, randomized phase II trial is underway of patients with unresectable or metastatic ACC randomized to EDP-mitotane with or without the progesterone analog megestrol acetate in the first-line setting (Table 2).^87^

## Relacorilant

Approximately half of ACC tumors secrete excess glucocorticoids, and hypercortisolism is correlated with worse survival in patients with ACC. Glucocorticoids exert an immunosuppressive effect through a broad array of mechanisms and are notably associated with T-cell depletion in ACC.^88,89^ This, in turn, is thought to contribute to ICI resistance.

Relacorilant is a glucocorticoid receptor modulator that is currently being investigated in the treatment of both Cushing syndrome and solid tumors.^90-93^ A phase Ib trial of combination pembrolizumab and relacorilant in patients with unresectable or metastatic ACC with excess glucocorticoid production is underway (Table 2).^94^

## Radiopharmaceuticals

Theranostic radiopharmaceuticals are compounds that are labeled with radionuclides and can be used for both diagnostic (i.e., imaging) and therapeutic purposes depending on the specific conjugated radionuclide: gamma and positron emitters for imaging, and beta-minus or alpha emitters for therapeutic targeting. They are in active use in a variety of cancers, notably in prostate cancer (prostate specific membrane antigen-targeted approaches) and neuroendocrine tumors (somatostatin receptor-targeted approaches), and under investigation in many others, including ACC.^95,96^ The utility of these agents is dependent on the robustness of target expression in tumor tissue.

Three trials of radiopharmaceuticals in ACC have been completed to date. Hahner et al^97^ studied iodine-123 metomidate (^123^I-IMTO) for PET/CT imaging and iodine-131 metomidate (^131^I-IMTO) for first-line or subsequent treatment of unresectable or metastatic ACC. Fourty-nine patients were enrolled, of whom 13 (27%) had tumors with very high ^123^I-IMTO uptake; 2 of these patients subsequently disenrolled from the study, and 1 patient died shortly after treatment with ^131^I-IMTO due to septic shock. Of the 10 patients treated with ^131^I-IMTO with follow-up imaging, 1 (10%) had PR and 5 (50%) had SD. The median PFS in these 6 patients was 14 months. ^131^I-IMTO was generally well-tolerated, with reversible bone marrow suppression being the most commonly observed adverse effect.

Grisanti et al^98^ studied gallium-68 dodecane tetraacetic acid Tyr3-octreotide (^68^Ga-DOTATOC) for PET/CT imaging and yttrium-90/lutetium-177 DOTATOC (^90^Y/^177^Lu-DOTATOC) for treatment of progressive metastatic ACC. 19 patients were enrolled, of whom 10 (53%) had tumors with ^68^Ga-DOTATOC uptake of any-grade intensity in metastatic sites, but only 2 of whom (11%) had tumors with strong uptake in a diffuse/multiple pattern that was deemed clinically significant. These 2 patients were treated with ^90^Y/^177^Lu-DOTATOC. One patient had PR that lasted 12 months, and other had SD that lasted 4 months. Mild back pain and grade 2 lymphopenia were observed in one patient.

Hahner et al^99^ later studied (R)-1-[1-(4-[iodine-123]iodophenyl)ethyl]-1H-imidazole-5-carboxylic acid azetidinyl amide (^123^I-IMAZA) for PET/CT imaging and iodine-131 IMAZA (^131^I-IMAZA) for treatment of progressive unresectable or metastatic ACC. 69 patients were enrolled, of whom 13 (19%) had tumors with strong uptake of ^123^I-IMAZA in all metastatic sites. All 13 patients were treated with ^131^I-IMAZA, and one was lost to follow-up. Of the 12 patients treated with ^131^I-IMAZA with follow-up imaging, 5 (42%) had SD. Median PFS was 4.1 months in the entire cohort, 14.3 months in the 5 patients who had SD, and 3.2 months in the 7 patients who had PD. ^131^I-IMAZA was generally well-tolerated, and, as with ^131^I-IMTO, reversible bone marrow suppression was the most commonly observed adverse effect.

A phase I trial of ^68^Ga-R8760, a radiopharmaceutical targeting the melanocortin 2 receptor (MC2R), for imaging of metastatic ACC is currently enrolling.^100^ To our knowledge, a counterpart therapeutic radiopharmaceutical targeting MC2R has not yet been developed.

Overall, the utility of radiopharmaceuticals in ACC is limited by the lack of robust target expression in most ACC tumors. However, for the subset of patients whose tumors do demonstrate robust expression, some promising results have been observed, and further investigation of these compounds is warranted. The additional benefit of a theranostic approach is that patients can effectively be screened with the diagnostic radiopharmaceutical for potential efficacy before undergoing treatment with the therapeutic counterpart, saving patients with poor target expression from a therapy that is unlikely to work. This screening mechanism is especially important in ACC due to its aggressive nature and poor survival.

## Vaccines

There is emerging interest in using therapeutic vaccines to treat ACC. One trial has been completed to date of the vaccine EO2401, which includes microbiome-derived peptides with molecular mimicry to 3 tumor-associated antigens known to be upregulated in ACC: IL13RA2, BIRC5, and FOXM1. Baudin et al^101^ studied EO2401 in combination with nivolumab in 33 patients with progressive (26 patients) or treatment-naïve (7 patients) metastatic ACC. Four patients (12%) had PR and 8 patients (24%) had SD. Median PFS was 1.9 months. The safety profile was consistent with nivolumab monotherapy except for a higher rate of injection site reactions.

A phase I trial is underway of the individualized mRNA neoantigen vaccine mRNA-0523-L001 for patients with advanced endocrine tumors, including ACC (Table 2).^102^

Overall, it remains to be seen whether therapeutic vaccines will have efficacy in treating ACC.

## CAR-T

Chimeric antigen receptor T (CAR-T) cells have an established role in the treatment of hematologic malignancies but remain under investigation for solid tumors. In ACC, several potential immunotherapeutic targets have been identified in preclinical studies, including B7H3, ROR1, and an antigen that yet to be disclosed.^103-105^ B7H3 may be of particular interest as a promising target, as B7H3 CAR-T cells have shown activity in children and young adults with solid tumors.^106^ A phase I trial of autologous B7H3 CAR-T cells in children with advanced solid tumors, including ACC, is underway (Table 2).^107^

## Conclusion

Unresectable or metastatic ACC remains a challenging disease with a poor prognosis for which more active therapies are being aggressively sought. In past eras, multiple single-agent chemotherapies and combination chemotherapy regimens were trialed, culminating in the establishment of the EDP-mitotane regimen as accepted first-line therapy. Over the past decade, attention has shifted to targeted and molecularly-defined approaches. Many of these approaches have been tested in clinical trials, almost all of which are early phase, with clinical data that have been reviewed here. Moreover, many more which have shown promise in preclinical studies have not yet entered clinical trials.

The body of clinical trials of nonchemotherapeutic approaches highlights the challenges of treating this aggressive disease. Across all trials reviewed here, objective responses were rare and most patients experienced disease progression. However, rates of SD were nontrivial in some trials, and some patients with an objective response or SD enjoyed a duration of response of months or even years. The presence of these rare encouraging outcomes underscores the need for better patient selection for novel therapies and the development of robust biomarkers to guide management decisions and predict prognosis.

It has been difficult for most ACC trials to meet ORR, PFS, or OS primary endpoints owing to small sample sizes and high rates of nonresponse (even EDP-mitotane did not improve OS over single-agent streptozocin). Accordingly, there is interest in developing additional secondary endpoints in ACC trials, and it is our view that even SD can be clinically significant in ACC due to its very short median OS.

While many of the approaches reviewed here have demonstrated negative results, others have yielded encouraging signals. More work remains to be done to build on these findings and develop effective novel therapies for this devastating disease.
